# Construction and Analysis of High-Complexity Ribosome Display Random Peptide Libraries

**DOI:** 10.1371/journal.pone.0002092

**Published:** 2008-05-21

**Authors:** Li-Min Yang, Jing-Lin Wang, Lin Kang, Shan Gao, Yan-hua Liu, Ting-Mao Hu

**Affiliations:** 1 State Key Laboratory of Pathogen and Biosecurity, Institute of Microbiology and Epidemiology, Academy of Military Medical Sciences, Beijing, China; 2 The Center for Molecular Virology, Institute of Microbiology, Chinese Academy of Sciences, Beijing, China; Baylor College of Medicine, United States of America

## Abstract

Random peptide libraries displayed on the ribosome are becoming a new tool for the *in vitro* selection of biologically relevant macromolecules, including epitopes, antagonists, enzymes, and cell-surface receptors. Ribosome display is a cell-free system of coupling individual nascent proteins (phenotypes) to their corresponding mRNA (genotypes) by the formation of stable protein-ribosome-mRNA complexes and permitting the selection of a functional nascent protein by iterative cycles of panning and reverse transcription-polymerase chain reaction (RT-PCR) amplification *in vitro*. The complexity of the random peptide library is critical for the success of a panning experiment; greater the diversity of sequences within the library, the more likely it is that the library comprises sequences that can bind a given target with specific affinity. Here, we have used the cell-free system *Escherichia coli* S30 lysate to construct high-complexity random peptide libraries (>10^14^ independent members) by introducing strategies that are different from the methods described by Mattheakis *et al.* and Lamla *et al.* The key step in our method is to produce nanomole (nmol) amounts of DNA elements that are necessary for *in vitro* transcription/translation by using PCR but not plasmid DNA. Library design strategies and protocols that facilitate rapid identification are also presented.

## Introduction

Since the mid-1980s, a number of different methods have been developed to screen peptide or protein libraries for specific binders. A majority of these methods, such as phage display [1], cell surface display [2], [3], plasmid display [4], and the yeast two-hybrid system [5], are so-called *in vivo* systems because living cells are involved in these processes of library generation or screening. Therefore, the complexities of such libraries are limited to about 10^9^ by transformation efficiency. This limitation could be resolved for *in vitro* display technologies such as ribosome [6], [7], [8] and mRNA displays [9] by introducing a cell-free translation system. Since this system does not require transformation, large libraries can be prepared and utilized for selection. Furthermore, diversifications can be conveniently introduced in this method, thus making evolutionary approaches easily accessible. Ribosome display is an *in vitro* technology for the simultaneous selection and evolution of proteins from diverse libraries. This technology relies on non-covalent ternary polypeptide-ribosome-mRNA complexes, which ensures the coupling of genotypes and phenotypes. The complexes lack a stop codon at the mRNA level, thus preventing the release of the mRNA and the polypeptide from the ribosome. High concentrations of magnesium and low temperature further stabilize the ternary complexes. These complexes, which are formed during *in vitro* translation, can directly be used to select for the properties of the displayed protein. The upper boundary on pool complexity with these libraries results from limits on PCR volumes that can be reasonably used to generate the library (0.1–1.0 L). Thus, this suggests that if methods to perform protein selection entirely *in vitro* could be developed, the transfection limitation would be overcome and constructing protein libraries as large as ∼10^15^ sequences might be possible.

Here, we comprehensively report the construction of high-complexity random peptide libraries with 1.2×10^14^ independent members based on the scaffold of amino acids T20–V109 of protein D (pD), a structured part of the capsid protein from phage Lambda, by using *in vitro* ribosome display technology. Increasing library size provides 2 key advantages: it improves the likelihood of extremely rare sequences being isolated, and it increases the diversity of sequences isolated in a given selection. Thus, it provides the foundation for the discovery of biologically relevant macromolecules, including epitopes, antagonists, enzymes, and cell-surface receptors, from high-complexity random peptide libraries.

## Results and Discussion

### Generation of the library

**Figure 1:**
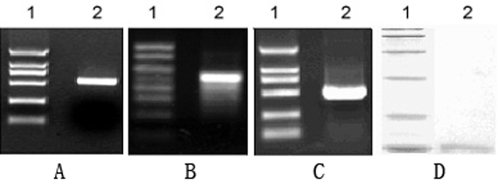
Construction of the random peptide library by ribosome display. (A) Preparation of the DNA template for ribosome display. Lane 1 is DL2000 DNA marker (2000, 1000, 750, 500, 250, 100 bp). Lane 2 is the full-length DNA template for ribosome display. (B) Identification of the mRNA product obtained by *in vitro* transcription. Lane 1 is RL1000 RNA marker (1000, 800, 600, 500, 400, 300, 200, 100 bp). Lane 2 is mRNA product. (C) Identification of the RT-PCR product. Lane 1 is DL2000 DNA marker. Lane 2 is RT-PCR product. (D) Identification of the protein product obtained by *in vitro* translation. Lane 1 is prestained protein marker (97.4, 66.2, 43, 31, 20.1, 14.4 kDa). Lane 2 is the full-length protein product.

**Figure 2:**
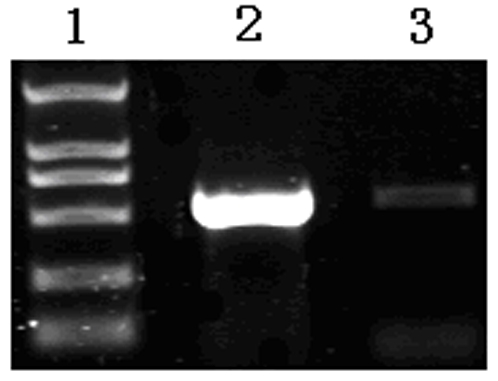
Identification of the ternary complexes formed during *in vitro* translation. Lane 1 is DL2000 DNA marker. Lane 2 is the ternary complexes that were treated with EDTA released the mRNA; the complexes were subsequently detected by RT-PCR. The RT-PCR product is indicated by an arrow. Lane 3 is the ternary complexes, which were not treated with EDTA, did not release the mRNA.

**Figure 3:**
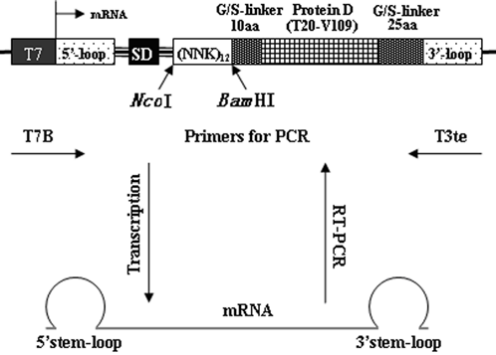
Description of the random DNA library construct used for ribosome display. The T7 promoter is followed by a SD sequence and the 12 random peptides; this is then followed sequentially by a 10-amino-acid G/S linker, T20–V109 of pD, and a 25-amino-acid G/S linker without a stop codon. Sequences encoding RNA stem-loop structures are present at both ends.

A DNA library encoding amino acids T20–V109 of protein D (pD) (a structured part of the capsid protein from phage Lambda, possessing 12 randomized amino acid residues at its N terminus) was generated by introducing the elements necessary for its efficient *in vitro* transcription/translation to a DNA fragment encoding 12 NNK codons through DNA ligation. The correct ligation product was a 543-bp fragment, which was purified by agarose gel electrophoresis (Figure 1A). The number of recovered DNA fragments directly reflected the complexity of the produced libraries which was up to 1.2×10^14^ members. The codons were designed to be NNK, where N represents equimolar G, A, T, or C, and K represents equimolar G or T. There are 32 possible codons resulting from the NNK motif: unique codons encoding 12 amino acids, 2 codons encoding 5 amino acids each, 3 codons encoding 3 amino acids each, and 1 stop codon (amber). In order to check the quality of the library, a small amount of a part of the 543-bp fragment was cloned, and 15 randomly chosen clones were sequenced. As expected, all the chosen clones were different (Figure 2). Five clones (one-third) exhibited a stop codon, and therefore, the library size was reduced to 8×10^13^ functional molecules (two-thirds of the original library). As shown in Figure 3, the construct used for ribosome display contained all necessary features: the T7 promoter at the DNA level, and the 5′ and 3′ stem-loops stabilizing the mRNA against ribonucleases at the RNA level as well as the Shine–Dalgarno (SD) sequence for efficient *in vitro* translation. The 5′-untranslated region of the mRNA was derived from gene 10 of phage T7 and was capable of forming a stable stem-loop structure. The 3′ stem-loop came from the early terminator of phage T3. The protein-coding sequence comprised a random peptide library and a spacer that tethers the synthesized protein to the ribosome. Till date, either a portion of the sequence from gene III from the phage M13mp19 [10] or a portion of the helical region of tolA [11] or the extended region of tonB [12] from *E. coli* has been used as a spacer sequence. We chose amino acids T20–V109 of protein D (pD), a structured part of the capsid protein from phage Lambda [13], as a spacer sequence. It has been shown that pD was stable, soluble, and highly expressed in *E. coli* and was monomeric in solution [14]. Further, T20–V109 of pD has been shown to be more resistant to proteolytic digestion than the full-length molecule while retaining the biophysical properties of the full-length construct. The pD sequence was followed by a 25-amino acid G/S linker, which was presumed to remain in the ribosomal tunnel when ternary complexes were formed.

This technique we reported has two main advantages over most other ribosome display reports. First, to compare with the traditional ribosome display technique, our technique is much easier and more convenient to construct the library since all the elements of the library construction were prepared by PCR, so it's very easy to complete constructing of a 10^14^ library in only 2 days. However, as the description of the traditional technique [13], it needs to insert all the random nucleotide sequence into the plasmid, we can conclude that construct a 10^14^ nucleotide library needs at least 200 mg plasmid, this is really a huge tough work, very difficult to make it. So, comparing with the reported methods of libraries construction, our technique is the most simple and efficiency one, it is convenient to be applied. Second, in the construct used for ribosome display, either a portion of the sequence from gene III from the phage M13mp19 or bovine heart fatty acid-binding protein (FABP) have been used as a C-terminal tether. However, we chose amino acids T_20_–V_109_ of protein D (pD), a structured part of the capsid protein from phage Lambda, as a spacer sequence. pD has been shown to be stable, soluble and resistant to proteolytic digestion. So the library we constructed is more stble than eaerly reported. In conclution, our technique is the most simple and efficiency one, and the library we constructed is the most stable one up to now.

### In vitro transcription and translation

The library was used for *in vitro* transcription, and the transcripts were analyzed by agarose gel electrophoresis. As expected, the 509-bp mRNA product was visualized by ethidium bromide staining (Figure 1B). The mRNA product was diluted and RT-PCR was performed with the primers T7B and T3te. The PCR product was analyzed by agarose gel electrophoresis (Figure 1C); a part of it was cloned, and 5 randomly chosen clones were sequenced. As expected, all the selected clones were different (data not shown). The efficacy of the *in vitro* translation system was tested by adding Transcend™ tRNA-containing biotinylated lysine into the mixture for *in vitro* translation; synthesized polypeptides were biotinylated during the process of translation. As visualized using the AP conjugate of avidin, a fusion protein containing a random polypeptide with a relative mass of 14 kDa was detected by Western blot (Figure 1D).

The ternary complexes, which were formed during *in vitro* translation, were stabilized by high concentrations of magnesium ions and low temperature. The mRNA incorporated into the bound ribosomal complexes can be eluted by the addition of EDTA and subsequently detected by RT-PCR. As expected, the ternary complexes, which were treated with EDTA, released mRNA and were subsequently detected by RT-PCR and agarose gel electrophoresis (Figure 2).

In *E. coli*, peptides synthesized from mRNAs without stop codons are modified by the addition of an 11-amino-acid peptide tag (AANDENYALAA) at the carboxy terminal. This peptide tag is encoded by 10S-RNA, the product of the ssrA gene. Addition of the peptide tag to the mRNA leads to its degradation by specific carboxy-terminal proteases in the cytoplasm and periplasm. The process of tagging an mRNA without a stop codon by 10S-RNA also occurs during *in vitro* translation. Therefore, we decided to block the 10S-RNA system by means of an antisense oligonucleotide (anti-ssrA) (Table 1) that was complementary to the tag-encoding sequence of 10S-RNA [15].

**Table 1:** Synthesized oligonucleotides for the construction of plasmid pT7PD and ribosome display library.

Oligonucleotides	Sequences (from 5′ end to 3′ end)
1. T7B	ATACGAAATTAATACGACTCACTATAGGGAGACCACAACGG
2. SDA	AGACCACAACGGTTTCCCTCTAGAAATAATTTTGTTTAACTTTAAGAAGGAGATATA
3. Linker	TCCACCTGCAGCGAAAAAGTAAAAAAGCCATGGATATATCTCCTTCTT
4. PD_up	GCTGCAGGTGGATCCGGCAGCGGTAGCGGCACCGCAACCGC
5. PD_down	GGCCCACCCGTGAAGGTGAGCCAACGATGCTGATTGC
6. Lib	AAGGAGATATATCCATGGCT (NNK)_12_ GGTGGAGGTGGATCCGG
7. G/S linker	ACCCGACCCACTTCCTGATCCCCCGCCACCGCTGCCACCCCCGCCTGATCCCCCGCCACCGCTGCCACCCCCGCCAACGATGCTGATTGC
8. T3te	GGCCCACCCGTGAAGGTGAGCCACCCGACCCACTTCC
9. lib_up	GCGAATTCATACGAAATTAATACGA
10. lib_down	ATAAGCTTGGCCCACCCG
11. anti-ssrA	CTTAAGCACACCAGTAAGGTGTGCGGTCAGGATATTCACCACAATCCC
12. librev	GTTGATGTCCGGATCCACCTCCACC

### Sequence analysis

**Figure 4:**
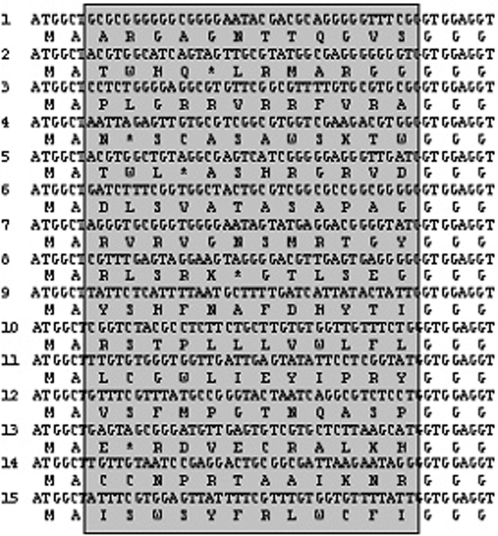
Alignment of the random nucleotide sequences and the predicted amino acid sequence of 15 randomly chosen clones. The random nucleotide sequences of these clones were different, and 5 clones (one-third) exhibited a stop codon.

**Figure 5:**
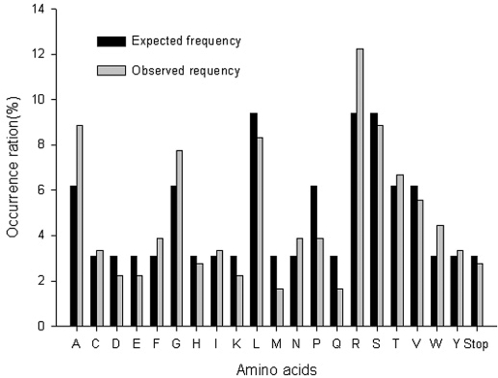
Distribution and occurrence ratio of the predicted amino acid sequence of the library. Occurrence ratio = amino acid occurrence÷total number of amino acids×100%. Expected frequency = number of codons for a particular amino acid÷32 codons×100%.

The sequence diversity of the library was estimated by sequencing 15 clones, translating each sequence, and comparing the observed frequency of each amino acid with that expected from the randomization scheme used to construct the library. When NNK was used to encode each random position, the expected frequency was equal to the number of codons for that amino acid÷32 codons×100%, or 9.4% for Arg, Leu, and Ser; 6.2% for Ala, Gly, Pro, Thr, and Val; and 3.1% for each of the remaining residues. The occurrence ratios of 20 amino acids and the stop codon were calculated, and no apparent amino acid bias was observed (Figure 4, 5).

In general, the chance of finding a particular activity within a library is proportional to the size of the library that can be screened. Lancet *et al.* [16], [17] predicted that increasing library size 10,000-fold should increase the best binding affinity found by more than 300-fold. In this study, we have successfully constructed high-complexity random 12-mers peptide libraries by using ribosome display technology. The size of the peptide libraries was up to 1.2×10^14^. Recently, Lamla *et al.* [18] constructed a synthetic library based on the scaffold of bovine heart fatty acid-binding protein (FABP) with 1.1×10^15^ independent members using ribosome display. During its construction, the DNA library, which encodes the random 15-mers peptide libraries, was generated by introducing a DNA fragment (5.4 nmol = 186 µg) encoding 15 NNS codons into the pFALinklpp vector (5.3 nmol = 13.5 mg) through DNA ligation. After ligation, the product was cleaved with *Hin*dIII and *Bam*HI, and subsequently used for *in vitro* transcripton and translation. It is common knowledge that it is very difficult to obtain 13.5 mg of plasmid DNA (vector); besides, the inefficiency of the vector looping during the ligation reaction is an additional limitation; hence, only a small amount of the final product is obtained. Here, we have improved this method by using PCR to produce nmol quantities of the DNA elements necessary for *in vitro* transcription and translation, and after ligation, the purified ligation product could be directly used for *in vitro* transcription and translation; thus, the reaction involving the use of restriction enzymes was avoided. Because nmol quantities of the DNA fragment can be conveniently produced using PCR, this method is comparatively more convenient and efficient. We envision that ribosome display technology will have a great impact on applications in biotechnology, medicine, and proteomics [19].

## Materials and Methods

### Main materials

All primers were synthesized by Bioasia Co. Ltd (Shanghai, China). The T7 RiboMAX™ Express Large Scale RNA Production System, *Escherichia coli* (*E. coli*) S30 Extract System, Transcend™ Non-Radioactive Translation Detection Systems, Wizard SV GEL, PCR Clean-Up System and Access reverse transcription polymerase chain reaction (RT-PCR) kits, RNase-free DNase I, and Pfu DNA polymerase were obtained from Promega (USA). The restriction enzymes *Nco*I, *Eco*RI, *Hin*dIII, and *Bam*HI were purchased from NEB (USA). The pMD18-T vector and T4 DNA ligase were obtained from TaKaRa Biotechnology Co. Ltd (Dalian, China). The RNeasy® MinElute™ Cleanup kit was purchased from Qiagen (Germany).

### Construction of plasmid pT7PD by overlapping PCR

Overlapping PCR was performed in 3 steps. (1) The primers T7B, SDA and the linker (Table 1) were mixed and assembled as follows: 2 min at 94°C, followed by 20 cycles of 30 s at 94°C, 30 s at 50°C, and 30 s at 72°C. The product was then purified from the gel. (2) The primers PD_up, PD_down, and phage Lambda (Table 1) were mixed and amplified as follows: 2 min at 94°C, followed by 25 cycles of 30 s at 94°C, 30 s at 30°C, and 30 s at 72°C. The product was then purified from the gel. (3) The purified products obtained from the first 2 steps and primers lib_up, lib_down, Gly/Ser (G/S) linker, and T3te (Table 1) were mixed and assembled as follows: 2 min at 94°C, followed by 25 cycles of 30 s at 94°C, 30 s at 50°C, and 50 s at 72°C; the PCR product was purified from the gel using the PCR Clean-Up System. The purified product was cleaved with *Eco*RI and *Hin*dIII and then cloned into plasmid pUC18. The resulting plasmid was termed pT7PD and verified by sequencing. It contained all the elements necessary for efficient *in vitro* transcription/translation and could be used for ribosome display.

### Construction of the ribosome display library

The plasmid pT7PD was used to generate DNA templates by PCR amplification with specifically designed oligonucleotide primers lib_up and lib_down using Pfu DNA polymerases according to the supplier's instructions. The PCR product was purified and cleaved with *Nco*I and *Bam*HI and separated by agarose gel electrophoresis. The 395-bp and 96-bp fragments were excised and purified. In order to generate the library, the oligonucleotide Librev was hybridized to the randomized oligonucleotide Lib (Table 1), and complementary strand synthesis was performed using Pfu DNA polymerase. The double-stranded product was cleaved with *Nco*I and *Bam*HI and separated by agarose gel electrophoresis. The 53-bp library fragment was excised and purified. The 3 purified fragments [53-bp (1 nmol = 35 µg), 395-bp (1.5 nmol = 385 µg), 96-bp fragments (1.5 nmol = 93.5 µg)] were ligated in a 5-ml reaction mixture with 10,500 units of phage T4 DNA ligase (TaKaRa) for 36 h at 4°C. After ligation, the whole reaction mixture was separated by agarose gel electrophoresis. The 543-bp fragment was excised and purified using the Gel and PCR Clean-Up System (Promega). This 543-bp fragment included all the elements necessary for transcription and translation and was analyzed by agarose gel electrophoresis. Further, its concentration was determined by measuring UV absorption; the amount of the purified fragment was 72 µg, which corresponded to 1.2×10^14^ molecules. A portion of this DNA fragment was cloned into the pMD18-T vector (TaKaRa) and transformed into calcium-competent *E. coli* TOP10 cells; 15 random clones were then sequenced.

### In vitro transcription

The library was directly used for *in vitro* transcription using T7 RNA polymerase (Promega) according to the supplier's instructions. The reaction mixtures were incubated for 1 h at 37°C. Following *in vitro* transcription, the reaction mixture was treated with 0.2 U/Wl RNase-free DNase I (Promega) for 15 min at 37°C. The transcripts were purified using the RNeasy clean up kit (Qiagen). Subsequently, they were analyzed by agarose gel electrophoresis, and their concentrations were determined by measuring UV absorption.

### RT-PCR

The transcripts were diluted 1∶5,000 with RNase-free H_2_O and RT-PCR was performed using Access RT-PCR System (Promega) according to the supplier's recommendation with the primers T7B and T3te. The PCR product was analyzed by agarose gel electrophoresis and purified from the gel; it was then cloned into the pMD18-T vector (TaKaRa) and transformed into calcium-competent *E. coli* TOP10 cells; 5 random clones were sequenced.

### In vitro translation

The *in vitro* translation reaction was based on an *E. coli* S30 lysate (Promega) and was performed according to the supplier's protocol with slightly modification. The *in vitro* translation for ribosome display was carried out in the presence of 5 µM anti-ssrA oligonucleotide and 30 µg mRNA as template, and ε-labeled biotinylated lysine-tRNA complex (Transcend™ tRNA, Promega) was added to the translation reaction system. After sodium dodecyl sulfate-polyacrylamide gel electrophoresis (SDS-PAGE) and electroblotting, the biotinylated proteins were visualized by colorimetric detection following binding by streptavidin-alkaline phosphatase (streptavidin-AP).

To measure the ternary complexes formed during the *in vitro* translation, we determined the fraction of total mRNA that was released from the ternary complexes after treatment with ethylendiaminotetraacetic acid (EDTA) [1]. Following an 8 min translation reaction at 37°C, the reaction was arrested by a 5-fold dilution with ice-cold wash buffer (50 mM Tris-acetate, pH 7.5; 150 mM NaCl; 50 mM magnesium acetate; 500 mM KCl). Equal amounts of the diluted reaction mixtures were centrifuged at 280,000 *g* for 35 min, 4°C, and the pellets were resuspended in wash buffer or elution buffer (50 mM Tris-acetate, pH 7.5; 150 mM NaCl; 20 mM EDTA) and centrifuged the second time at 280,000 *g* for 35 min, 4°C. The supernatant was purified using the RNeasy clean up kit (Qiagen), and RT-PCR was performed with the primers T7B and T3te. PCR product was analyzed by agarose gel electrophoresis.
