# Correction: Construction and Analysis of High-Complexity Ribosome Display Random Peptide Libraries

**DOI:** 10.1371/annotation/b2f256d4-ba3d-41d3-b30a-069f5734a82c

**Published:** 2012-08-06

**Authors:** Li-Min Yang, Jing-Lin Wang, Lin Kang, Shan Gao, Yan-hua Liu, Ting-Mao Hu

When published on May 21, 2008, the first four sentences in the Introduction section and several sentences under the Results and Discussion section were copied verbatim from the article below -reference 18 in the article:

J Mol Biol. 2003 May 30;329(2):381-8.Searching sequence space for high-affinity binding peptides using ribosome display. Lamla T, Erdmann VA.

The authors are therefore issuing this Correction to make readers aware of this overlap in text and to apologize to Lamla and Erdmann, the readers and the editors for this inadequate use of text from the previous article.

